# Associations of 25 Hydroxyvitamin D and High Sensitivity C-reactive Protein Levels in Early Life

**DOI:** 10.3390/nu14010015

**Published:** 2021-12-21

**Authors:** Nicklas Brustad, Nadia R. Fink, Jakob Stokholm, Klaus Bønnelykke, Nilofar V. Følsgaard, David Hougaard, Susanne Brix, Jessica Lasky-Su, Scott T. Weiss, Bo Chawes

**Affiliations:** 1 COPSAC, Copenhagen Prospective Studies on Asthma in Childhood, Herlev and Gentofte Hospital, University of Copenhagen, 2820 Copenhagen, Denmark; nicklas.brustad@dbac.dk (N.B.); rahmanfink@gmail.com (N.R.F.); stokholm@copsac.com (J.S.); kb@copsac.com (K.B.); nilo.foelsgaard@dbac.dk (N.V.F.); 2 Section for Clinical Mass Spectrometry, Danish Center for Neonatal Screening, Department of Congenital Disorders, Statens Serum Institut, 2300 Copenhagen, Denmark; DH@ssi.dk; 3 Department of Biotechnology and Biomedicine, Technical University of Denmark, 2800 Kongens Lyngby, Denmark; sbrix@dtu.dk; 4 Channing Division of Network Medicine, Brigham and Women’s Hospital and Harvard Medical School, Boston, MA 02115, USA; rejas@channing.harvard.edu (J.L.-S.); restw@channing.harvard.edu (S.T.W.)

**Keywords:** vitamin D, hs-CRP, low-grade inflammation, COPSAC, 25(OH)D, pregnancy, children

## Abstract

Vitamin D deficiency and elevated high sensitivity C-reactive protein (hs-CRP) have been associated with several health outcomes, but knowledge on early life trajectories and association between 25 hydroxyvitamin D (25(OH)D) and hs-CRP is lacking. We investigated the association between longitudinal measurements of 25(OH)D and hs-CRP, respectively, from pregnancy to childhood and throughout childhood in two Danish mother–child cohorts—the COPSAC_2010_ and COPSAC_2000_. In COPSAC_2010_, there was an association between 25(OH)D concentrations at week 24 in pregnancy and at age 6 months in childhood (*n =* 633): estimate (95% CI); 0.114 (0.041;0.187), *p =* 0.002, and between 25(OH)D at age 6 months and 6 years (*n =* 475): 0.155 (0.083;0.228), *p <* 0.001. This was also demonstrated in the COPSAC_2000_ cohort between 25(OH)D concentrations in cord blood and at age 4 years (*n =* 188): 0.294 (0.127;0.461), *p <* 0.001 and at age 6 months and 4 years (*n =* 264): 0.260 (0.133;0.388), *p <* 0.001. In COPSAC_2000_, we also found an association between hs-CRP at age 6 months and 12 years in childhood (*n =* 232): 0.183 (0.076;0.289), *p <* 0.001. Finally, we found a negative association between the cross-sectional measurements of 25(OH)D and hs-CRP at age 6 months (*n =* 613) in COPSAC_2010_: −0.004 (−0.008;−0.0004), *p =* 0.030, but this was not replicated in COPSAC_2000_. In this study, we found evidence of associations across timepoints of 25(OH)D concentrations from mid-pregnancy to infancy and through childhood and associations between hs-CRP levels during childhood, although with weak correlations. We also found a negative cross-sectional association between 25(OH)D and hs-CRP concentrations in COPSAC_2010_ proposing a role of vitamin D in systemic low-grade inflammation, though this association was not present in COPSAC_2000_.

## 1. Introduction

High-sensitivity C-reactive protein (hs-CRP) is a known marker of systemic low-grade inflammation in many chronic disorders, including inflammatory bowel disease (IBD) [1], cardiovascular disease [2,3], depression [4] and chronic obstructive pulmonary disease (COPD) [5]. Further, increased concentrations of hs-CRP have been linked to decreased lung function in childhood [6,7], allergic sensitization at school age [8], early life airway microbiota [9] and childhood asthma [10,11], which has led to suggestions of using hs-CRP as a clinical biomarker of low-grade inflammation for grading, diagnosing and preventing disease [11]. We have previously shown an association between hs-CRP levels in pregnant mothers and their offspring at age 6 months [12]; however, knowledge on the development of low-grade inflammation throughout childhood is lacking.

Vitamin D sufficiency in early life has also been shown to be associated with several health outcomes during childhood, including greater bone mineralization [13], decreased risk of enamel defects [14], asthma [15] and various skin conditions. Experimental studies have suggested reduced replication of virus in bronchial epithelial cells [16], induced antimicrobial production [17] and upregulation in the early life airway immune profile [18] as possible mechanisms for preventing asthma. Since the cutaneous conversion of 7-dehydrocholesterol to pre-vitamin D_3_ and then vitamin D_3_ occurs only when exposed to sunlight by ultraviolet B radiation [19], human blood concentrations depend on many factors such as pigmentation, lifestyle, skin protection, etc. It is unclear whether vitamin D status remains stable from early to later in life, but it has previously been demonstrated in the Western Australian Pregnancy Cohort (Raine) longitudinal study that 25 hydroxyvitamin D (25(OH)D) concentrations tracked from school age until age 20 years [20]. However, the Raine study did not investigate the relationship between maternal vitamin D concentrations during pregnancy and vitamin D concentrations through early childhood.

In this study, we utilized two Danish mother–child cohorts—the Copenhagen Prospective Studies on Asthma in Childhood (COPSAC) 2000 and 2010—to investigate potential association across timepoints of 25(OH)D and hs-CRP concentrations during pregnancy and childhood and examined the possible relationship between 25(OH)D and hs-CRP, which are important for childhood health and disease.

## 2. Materials and Methods

### 2.1. Ethics

The studies were approved by the local Ethics Committee (HKF 01-289/96; H-B-2008-093) and the Danish Data Protection Agency (2015-41-3696). Both oral and written informed consent was obtained from the parents during enrollment.

### 2.2. Study Populations

The Danish COPSAC_2000_ and COPSAC_2010_ clinical, single-center, mother–child cohorts have previously been described in detail including enrollment procedure, baseline characteristics and flow of the participants [13,18,21,22].

In summary, the prospective COPSAC_2000_ cohort is a high-risk asthma cohort of 411 children born to mothers with a history of asthma, which were enrolled during pregnancy at week 36. The children were monitored from age 1 month until age 18 years undergoing a minimum of 18 scheduled and acute care clinical visits [22,23], allowing for deep phenotyping of the children.

The COPSAC_2010_ is a population-based cohort including 700 children of pregnant mothers enrolled at week 24 in pregnancy. The pregnant women participated in two randomized controlled trials of high-dose (2800 IU/day) vs. standard-dose (400 IU/day) vitamin D [18] and fish-oil vs. olive-oil [24] from week 24 gestation until 1 week postpartum. The children were followed longitudinally in the COPSAC research clinic with a minimum of 14 scheduled and acute care visits from age 1 week until age 10 years.

### 2.3. Measurements of Hs-CRP and 25(OH)D

In COPSAC_2000_, blood samples from the cubital vein of the children at age 6 months, 7 and 12 years were centrifuged and stored at −80 °C until analysis, where hs-CRP concentrations were determined by a high-sensitivity electrochemiluminescence assay from MesoScale Discovery with a lower limit of detection of 0.007 ng/mL. Total serum 25(OH)D concentrations were measured at birth in cord blood and at 4 years of age using the isotope dilution liquid chromatography–tandem mass spectrometry [25]. Total plasma 25(OH)D concentrations were measured at age 6 months using the same technique as above. The laboratories participated in the proficiency testing program Vitamin D External Quality Assessment Scheme (DEQAS).

In COPSAC_2010_, blood samples from the children at age 6 months were analyzed for hs-CRP concentrations using a similar method as in COPSAC_2000_. Total serum 25(OH)D concentrations were measured from maternal blood samples in pregnancy week 24 using the same method as above. Child samples at age 6 months and 6 years were analyzed using the DiaSorin LIAISON 25-OH Vitamin D Total Assay [26]. The laboratory used US National Institute of Standards and Technology (NIST) level 1 protocol.

### 2.4. Covariates

We included environmental determinants previously shown to be related to hs-CRP and 25(OH)D concentrations in the children from our cohorts [6,23,27], which were sex, season of samples, older children in home at birth and any infection 14 days prior to hs-CRP measurement based on daily diary registrations of symptoms of cold, cough, pneumonia, ear infection, fever or gastric infection [22].

### 2.5. Statistical Analyses

The analyses of the associations between hs-CRP and 25(OH)D at different timepoints were performed using linear regression models and illustrated by scatter plots. Additionally, the models were adjusted for covariates. The hs-CRP values were log-transformed prior to analyses, given the skewed distribution of data. All analyses were performed using R (version 4.0.3) with *p <* 0.05 considered indicative of significance.

## 3. Results

### 3.1. Associations of 25(OH)D from Pregnancy to Childhood and in Childhood

**Figure 1:**
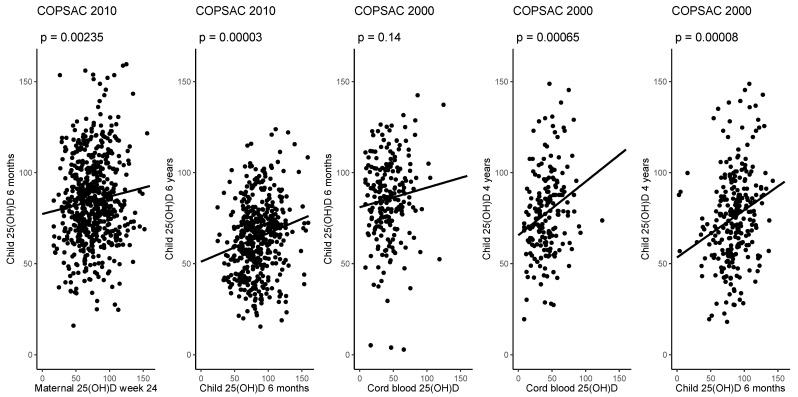
The associations between 25(OH)D at pregnancy week 24, age 6 months and 6 years in COPSAC_2010_ and cord blood, age 6 months and 4 years in childhood in COPSAC_2000_. All values are in nmol/L.

Of the 700 children in the COPSAC_2010_ cohort, 633 (90%) had available serum 25(OH)D measurements at age 6 months (mean (SD): 84.8 (23.8) nmol/L) with mothers with available 25(OH)D measurements at pregnancy week 24. We found an association between concentrations at week 24 in pregnancy and at age 6 months in childhood: crude estimate (95% CI); 0.114 (0.041;0.187), *p =* 0.002, although the correlation was weak (*R*^2^ = 0.015). At age 6 years, 475 (75%) of the children with 6 months measurements had available serum 25(OH)D measurements (mean (SD): 64.3 (20.0) nmol/L), which demonstrated an association between these two time points: 0.155 (0.083;0.228), *p <* 0.001, *R*^2^ = 0.036 (Figure 1).

Of the 411 children in the COPSAC_2000_ cohort, 257 (63%) had available cord blood measurements (mean (SD): 43.1 (20.8) nmol/L), 347 (84%) had available measurements at age 6 months (mean (SD): 85.9 (22.7) nmol/L) and 298 (73%) had available measurements at age 4 years of serum 25(OH)D (mean (SD): 76.0 (25.4) nmol/L). Among the 215 children with both cord blood and 6 months 25(OH)D measurements, we did not find a significant association between these time points: estimate (95% CI); 0.101 (−0.034;0.236), *p =* 0.143, *R*^2^ = 0.010; however, we found a significant association between 25(OH)D from cord blood and at 4 years during childhood (*n =* 188): 0.294 (0.127;0.461), *p <* 0.001, *R*^2^ = 0.061, and a significant association between 25(OH)D at age 6 months and 4 years (*n =* 264): 0.260 (0.133;0.388), *p <* 0.001, *R*^2^ = 0.058 (Figure 1).

### 3.2. Associations of hs-CRP in Childhood

**Figure 2:**
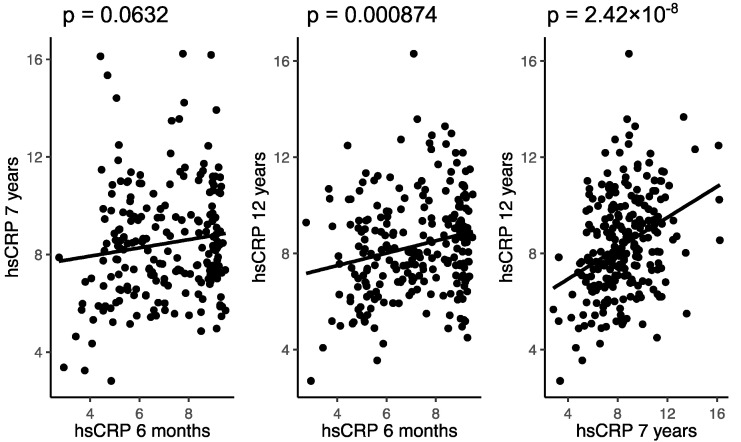
The associations between hs-CRP at age 6 months, 7 and 12 years in childhood in the COPSAC_2000_ cohort. All values are in ng/mL and log transformed.

Of the 411 children in COPSAC_2000_, 300 (73%), 276 (67%) and 313 (76%) had available hs-CRP measurements (ng/mL) at age 6 months, 7 and 12 years in childhood, respectively. Among the 211 children with hs-CRP measurements at both 6 months and 7 years, a trend towards an association was observed: crude estimate (95% CI); 0.097 (−0.005;0.200), *p =* 0.063, *R*^2^ = 0.016. In children (*n =* 232) with both 6 months and 12 years hs-CRP measurements, we found an association between these two time points: 0.183 (0.076;0.289), *p <* 0.001, *R*^2^ = 0.047, which was also significant in the analysis of children (*n =* 247) with hs-CRP at age 7 vs. 12 years: 0.373 (0.246;0.501), *p <* 0.001, *R*^2^ = 0.120 (Figure 2).

### 3.3. Association between Hs-CRP and 25(OH)D in Both Cohorts

Cross-sectional measurements of hs-CRP and serum 25(OH)D at age 6 months were available in 613 (88%) children in COPSAC_2010_. There was a negative association between hs-CRP and 25(OH)D from a linear regression model: crude estimate (95% CI); −0.004 (−0.008;−0.0004), *p =* 0.030. Among the 613 children with both hs-CRP and 25(OH)D measurements at age 6 months, 208 children with any diary registered infection 14 days prior to hs-CRP measurement were excluded in a stratified model, leaving 405 children available for analysis, which still showed a negative association between hs-CRP and 25(OH)D: −0.005 (−0.009;−0.0006), *p =* 0.027. However, in a fully adjusted analysis for sex, sample season, older children in home and any infections 14 days prior to measurement, we did not find an association (Table 1).

In COPSAC_2000_, 299 (73%) children had cross-sectional measurements of hs-CRP and plasma 25(OH)D at age 6 months with no significant association between these: 0.003 (−0.003;0.009), *p =* 0.401. In the fully adjusted model of sex, sample season, older children in home and any infection 14 days prior to measurement, there was still no significant association (*n =* 299): 0.004 (−0.002;0.010), *p =* 0.157. In children with no infection 14 days prior to measurement (*n =* 208), there was also no association: 0.003 (−0.004;0.009), *p =* 0.436 (Table 2).

## 4. Discussion

### 4.1. Primary Findings

In two Danish mother–child cohorts with close longitudinal follow-up, we found evidence of association across timepoints of serum 25(OH)D concentrations from mid-pregnancy to childhood and throughout childhood. Further, we found association between hs-CRP concentrations measured from early childhood at age 6 months through to age 12 years, suggesting an early trajectory of both 25(OH)D and hs-CRP. We demonstrated a negative association between hs-CRP and serum 25(OH)D concentrations using cross-sectional measurements at age 6 months in the COPSAC_2010_ cohort, proposing a role of vitamin D in systemic low-grade inflammation. However, the inverse association between hs-CRP and 25(OH)D was not apparent in the high-risk COPSAC_2000_ cohort.

### 4.2. Strengths and Limitations

The main strength of our study is the close longitudinal clinical follow-up of the children from two large-scale cohorts with several blood samples performed both in pregnancy and during childhood, which allows for analyses of correlation of measurements over a long period. Another strength is the thorough, deep phenotyping of the children with daily diary cards filled out by the parents in COPSAC_2010_ with registration of any signs of infections, which is crucial when assessing hs-CRP given its well-established role as a marker of inflammation and infection. Additionally, there is information on a wide range of environmental and demographic exposures, which previously have been used for identification of determinants of hs-CRP and 25(OH)D concentrations [6,23,27]. A limitation of the study is the lack of information on other important factors for 25(OH)D concentrations such as sunscreen protection, hours spent in the sun and diet in both cohorts and although we adjusted for important covariates based on our previous studies, our findings could be influenced by residual lifestyle confounders given the observational study design. Another limitation is the lack of ethnic diversity in our cohorts consisting primarily of Caucasians, which only allows for generalization of our findings among this ethnic group and, therefore, may not be applicable to other populations. Finally, it was a limitation that our cohorts were not similar in terms of population characteristics and sample sizes, where the COPSAC_2010_ is a larger population-based cohort [21] and the COPSAC_2000_ is a smaller high-risk cohort [22], which may explain why we did not find an association between hs-CRP and 25(OH)D at age 6 months in COPSAC_2000_, despite adjusting for relevant covariates. The difference in measurement methods of 25(OH)D in the two cohorts could possibly also explain why the results differ when analyzing the relationship with hs-CRP; however, it should not influence the 25(OH)D correlations within the cohorts as the same method is used within each cohort.

### 4.3. Interpretation

Our findings of associations between 25(OH)D concentrations from pregnancy to childhood and through childhood until age 6 years are in line with previous studies among older populations [20,28]. In the Australian Raine study, 25(OH)D concentrations at age 6 years were associated with concentrations measured until age 20 years [20]. Further, 25(OH)D status at age 6 years was characterized as a predictor of peak bone mass around age 20 years in the same cohort, which highlights the clinical importance of early life vitamin D sufficiency, since our findings demonstrated an association across timepoints already from pregnancy week 24 to childhood. The association of 25(OH)D concentrations over time has also been shown in a Norwegian study over a 14-year period in adulthood [28], but was not found in a mixed South African population investigating correlation from age 11 to 20 years [29]. The latter study was limited by the number of subjects (*n =* 76) and could also reflect that the association over time is diverse across ethnic groups. The clinical importance of vitamin D status has been investigated in relation to several disorders, and low 25(OH)D concentration has been suggested to be related to increased risk of, e.g., bone, inflammatory and infectious diseases [30]. Most notably, the risk of osteoporosis seems dependent on child bone mineralization [31,32], which is suggested to be highly influenced by early life vitamin D status [13]. Further, supplementation with high doses of vitamin D in pregnancy has shown to protect against early asthma development, suggesting a role of vitamin D in asthma prevention.

The association between hs-CRP concentrations at age 6 months and age 12 years is also in line with previous literature [33,34]. The JUPITER study (*n =* 8901) demonstrated an association between hs-CRP concentrations measured over a 4-year period among a mixed ethnic population of adults [33]. This finding was supported by the Cardiovascular Risk in Young Finns Study where adulthood CRP was predicted by childhood measurements (*n =* 1617) during a 21-year follow-up [34]. The clinical implications of elevated hs-CRP have been investigated in relation to cardiovascular disease risk in particular and described as a predictor of coronary heart disease [3]. In addition, increased hs-CRP concentrations have been linked to a broad range of diseases, including inflammatory bowel disease (IBD) [1], depression [4], COPD [5], decreased lung function in childhood [6,7], allergic sensitization at school age [8], early life airway microbiota [9] and childhood asthma [10,11].

It was previously shown in the COPSAC_2010_ cohort that hs-CRP concentrations in the pregnant mother at week 24 of gestation were associated with concentrations at age 6 months [12], which adds to the hypothesis of association across timepoints of hs-CRP concentrations beginning in early life similar to the associations between 25(OH)D concentrations from pregnancy through childhood demonstrated in this paper. Interestingly, we also found that these two measures were negatively correlated at age 6 months in the COPSAC_2010_ cohort. A previous meta-analysis (*n =* 924) showed the beneficial effect of vitamin D supplementation (400–7143 IU/day) on hs-CRP concentrations across different populations and diseases, suggesting a protective effect of vitamin D against systemic low-grade inflammation, which is linked to the development of disease [35]. Considering the proposed role of vitamin D in the inflammatory response [36], this effect is biologically plausible, which indicates a protective role of maintaining sufficient circulating 25(OH)D concentrations to protect against low-grade inflammation and possibly protect against associated disorders such as cardiovascular disease.

## 5. Conclusions

We found significant associations between 25(OH)D concentrations from pregnancy to childhood and through childhood, and associations between hs-CRP concentrations through childhood, although with weak correlations. Further, we found a negative cross-sectional association between hs-CRP and 25(OH)D concentrations in early childhood, suggesting a role of vitamin D in systemic low-grade inflammation, though this association was not present in COPSAC_2000_. These findings could potentially lead to the development of new preventive strategies due to the established role of low-grade inflammation in many chronic disorders, which is reflected by concentrations of hs-CRP. As a result of the known immune modulatory effects of vitamin D and the observation of inverse association between 25(OH)D and hs-CRP in this study, supplementation with vitamin D may revert systemic low-grade inflammation and prevent the development of a broad range of health outcomes.

## Figures and Tables

**Table 1:** The association between log hs-CRP and vitamin D at age 6 months in COPSAC_2010_ from uni- and multivariable linear regression models.

	hs-CRP Estimate *	95% CI	*p* Value
Crude (*n =* 613)	−0.004	−0.008; −0.0004	0.030
Adjusted for environmental and demographic factors (*n =* 613) ^1^	−0.002	−0.006; 0.001	0.230
Children with no infection (*n =* 405) ^2^	−0.005	−0.009; −0.001	0.027

^1^ Environmental and demographic factors: Sex, sample season, older children in home and any infection 14 days prior to measurement. ^2^ Including children with no infections 14 days prior only. * CRP values are in ng/mL and log-transformed.

**Table 2:** The association between log hs-CRP and vitamin D at age 6 months in COPSAC_2000_ from uni- and multivariable linear regression models.

	hs-CRP Estimate *	95% CI	*p* Value
Crude (*n =* 299)	0.003	−0.003; 0.009	0.401
Adjusted for environmental and demographic factors (*n =* 299) ^1^	0.004	−0.002; −0.010	0.157
Children with no infection (*n =* 208) ^2^	0.003	−0.004; −0.009	0.436

^1^ Environmental and demographic factors: Sex, sample season, older children in home and any infection 14 days prior to measurement. ^2^ Including children with no infections 14 days prior only. * CRP values are log-transformed.

## Data Availability

Anonymized data available on request by mail to chawes@copsac.com with publication.

## References

[1] Hod K., Ringel-Kulka T., Martin C.F., Maharshak N., Ringel Y. (2016). High-sensitive C-Reactive Protein as a Marker for Inflammation in Irritable Bowel Syndrome. J. Clin. Gastroenterol..

[2] Fonseca F.A.H., Izar M.C.D.O. (2016). High-Sensitivity C-Reactive Protein and Cardiovascular Disease Across Countries and Ethnicities. Clinics.

[3] Danesh J., Wheeler J.G., Hirschfield G., Eda S., Eiriksdottir G., Rumley A., Lowe G.D., Pepys M.B., Gudnason V. (2004). C-Reactive Protein and Other Circulating Markers of Inflammation in the Prediction of Coronary Heart Disease. N. Engl. J. Med..

[4] Valkanova V., Ebmeier K.P., Allan C.L. (2013). CRP, IL-6 and depression: A systematic review and meta-analysis of longitudinal studies. J. Affect. Disord..

[5] Leuzzi G., Galeone C., Taverna F., Suatoni P., Morelli D., Pastorino U. (2017). C-reactive protein level predicts mortality in COPD: A systematic review and meta-analysis. Eur. Respir. Rev..

[6] Chawes B., Stokholm J., Bønnelykke K., Brix S., Bisgaard H. (2015). Neonates with reduced neonatal lung function have systemic low-grade inflammation. J. Allergy Clin. Immunol..

[7] Ko A.R., Kim Y.H., Sol I.S., Kim M.J., Yoon S.H., Kim K.W., Kim K.-E. (2016). High-Sensitivity C-Reactive Protein Can Reflect Small Airway Obstruction in Childhood Asthma. Yonsei Med. J..

[8] Chawes B.L., Stokholm J., Schoos A.M., Fink N.R., Brix S., Bisgaard H. (2017). Allergic sensitization at school age is a systemic low-grade inflammatory disorder. Allergy.

[9] Fink N.R., Chawes B.L., Thorsen J., Stokholm J., Krogfelt K.A., Schjørring S., Kragh M., Bønnelykke K., Brix S., Bisgaard H. (2018). Neonates colonized with pathogenic bacteria in the airways have a low-grade systemic inflammation. Allergy.

[10] Shimoda T., Obase Y., Kishikawa R., Iwanaga T. (2015). Serum high-sensitivity C-reactive protein can be an airway inflammation predictor in bronchial asthma. Allergy Asthma Proc..

[11] Deraz T., Kamel T.B., El-Kerdany T.A., El-Ghazoly H.M. (2011). High-sensitivity C reactive protein as a biomarker for grading of childhood asthma in relation to clinical classification, induced sputum cellularity, and spirometry. Pediatr. Pulmonol..

[12] Fink N.R., Chawes B., Bønnelykke K., Thorsen J., Stokholm J., Rasmussen M.A., Brix S., Bisgaard H. (2019). Levels of Systemic Low-grade Inflammation in Pregnant Mothers and Their Offspring are Correlated. Sci. Rep..

[13] Brustad N., Garland J., Thorsen J., Sevelsted A., Krakauer M., Vinding R.K., Stokholm J., Bønnelykke K., Bisgaard H., Chawes B.L. (2020). Effect of High-Dose vs Standard-Dose Vitamin D Supplementation in Pregnancy on Bone Mineralization in Offspring Until Age 6 Years. JAMA Pediatr..

[14] Nørrisgaard P.E., Haubek D., Kühnisch J., Chawes B.L., Stokholm J., Bønnelykke K., Bisgaard H. (2019). Association of High-Dose Vitamin D Supplementation During Pregnancy with the Risk of Enamel Defects in Offspring. JAMA Pediatr..

[15] Wolsk H.M., Chawes B.L., Litonjua A.A., Hollis B.W., Waage J., Stokholm J., Bønnelykke K., Bisgaard H., Weiss S.T. (2017). Prenatal vitamin D supplementation reduces risk of asthma/recurrent wheeze in early childhood: A combined analysis of two randomized controlled trials. PLoS ONE.

[16] Schögler A., Muster R.J., Kieninger E., Casaulta C., Tapparel C., Jung A., Moeller A., Geiser T., Regamey N., Alves M.P. (2015). Vitamin D represses rhinovirus replication in cystic fibrosis cells by inducing LL-37. Eur. Respir. J..

[17] Wang T.T., Nestel F.P., Bourdeau V., Nagai Y., Wang Q., Liao J., Tavera-Mendoza L., Lin R., Hanrahan J.W., Mader S. (2004). Cutting Edge: 1,25-Dihydroxyvitamin D3 Is a Direct Inducer of Antimicrobial Peptide Gene Expression. J. Immunol..

[18] Chawes B.L., Bønnelykke K., Stokholm J., Vissing N.H., Bjarnadóttir E., Schoos A.M., Wolsk H.M., Pedersen T.M., Vinding R.K., Thorsteinsdóttir S. (2016). Effect of Vitamin D3 Supplementation During Pregnancy on Risk of Persistent Wheeze in the Offspring: A Randomized Clinical Trial. JAMA.

[19] DeLuca H.F. (2004). Overview of general physiologic features and functions of vitamin D. Am. J. Clin. Nutr..

[20] Zhu K., Oddy W.H., Holt P., Ping-Delfos W.C.S., Mountain J., Lye S., Pennell C., Hart P.H., Walsh J.P. (2017). Tracking of vitamin D status from childhood to early adulthood and its association with peak bone mass. Am. J. Clin. Nutr..

[21] Bisgaard H., Vissing N.H., Carson C.G., Bischoff A.L., Følsgaard N.V., Kreiner E., Chawes B., Stokholm J., Pedersen S.B., Bjarnadóttir E. (2013). Deep phenotyping of the unselected COPSAC 2010 birth cohort study. Clin. Exp. Allergy.

[22] Bisgaard H. (2004). The Copenhagen Prospective Study on Asthma in Childhood (COPSAC): Design, rationale, and baseline data from a longitudinal birth cohort study. Ann. Allergy Asthma Immunol..

[23] Schoos A.-M.M., Vinther C., Nørgaard S., Brustad N., Stokholm J., Bønnelykke K., Bisgaard H., Chawes B.L. (2019). Environmental and Genetic Determinants of Serum 25(OH)-Vitamin D Levels during Pregnancy and Early Childhood. Children.

[24] Bisgaard H., Stokholm J., Chawes B., Vissing N.H., Bjarnadóttir E., Schoos A.-M.M., Wolsk H.M., Pedersen T.M., Vinding R.K., Thorsteinsdóttir S. (2016). Fish Oil–Derived Fatty Acids in Pregnancy and Wheeze and Asthma in Offspring. N. Engl. J. Med..

[25] Højskov C.S., Heickendorff L., Møller H.J. (2010). High-throughput liquid–liquid extraction and LCMSMS assay for determination of circulating 25(OH) vitamin D3 and D2 in the routine clinical laboratory. Clin. Chim. Acta.

[26] Ersfeld D.L., Rao D., Body J.-J., Sackrison J.L., Miller A.B., Parikh N., Eskridge T.L., Polinske A., Olson G.T., MacFarlane G.D. (2004). Analytical and clinical validation of the 25 OH vitamin D assay for the LIAISON^®^ automated analyzer. Clin. Biochem..

[27] Brustad N., Greve J.H., Mirzakhani H., Pedersen C.T., Eliasen A.U., Stokholm J., Lasky-Su J., Bønnelykke K., Litonjua A.A., Weiss S.T. (2021). High-dose vitamin D during pregnancy and pathway gene polymorphisms in prevention of offspring persistent wheeze. Pediatr. Allergy Immunol..

[28] Jorde R., Sneve M., Hutchinson M., Emaus N., Figenschau Y., Grimnes G. (2010). Tracking of Serum 25-Hydroxyvitamin D Levels During 14 Years in a Population-based Study and During 12 Months in an Intervention Study. Am. J. Epidemiol..

[29] Poopedi M.A., Norris S.A., Micklesfield L.K., Pettifor J.M. (2015). Does vitamin D status track through adolescence?. Am. J. Clin. Nutr..

[30] Zittermann A. (2003). Vitamin D in preventive medicine: Are we ignoring the evidence?. Br. J. Nutr..

[31] Weaver C.M., Gordon C.M., Janz K.F., Kalkwarf H.J., Lappe J.M., Lewis R., O’Karma M., Wallace T.C., Zemel B.S. (2016). The National Osteoporosis Foundation’s position statement on peak bone mass development and lifestyle factors: A systematic review and implementation recommendations. Osteoporos. Int..

[32] Cooper C., Walker-Bone K., Arden N., Dennison E. (2000). Novel insights into the pathogenesis of osteoporosis: The role of intrauterine programming. Rheumatology.

[33] Glynn R.J., MacFadyen J.G., Ridker P.M. (2009). Tracking of High-Sensitivity C-Reactive Protein after an Initially Elevated Concentration: The JUPITER Study. Clin. Chem..

[34] Juonala M., Viikari J.S., Rönnemaa T., Taittonen L., Marniemi J., Raitakari O.T. (2006). Childhood C-reactive protein in predicting CRP and carotid intima-media thickness in adulthood: The Cardiovascular Risk in Young Finns Study. Arterioscler. Thromb. Vasc. Biol..

[35] Chen N., Wan Z., Han S.-F., Li B.-Y., Zhang Z.-L., Qin L.-Q. (2014). Effect of Vitamin D Supplementation on the Level of Circulating High-Sensitivity C-Reactive Protein: A Meta-Analysis of Randomized Controlled Trials. Nutrients.

[36] Agrawal D., Yin K. (2014). Vitamin D and inflammatory diseases. J. Inflamm. Res..

